# Citrange Fruit Extracts Alleviate Obesity-Associated Metabolic Disorder in High-Fat Diet-Induced Obese C57BL/6 Mouse

**DOI:** 10.3390/ijms141223736

**Published:** 2013-12-05

**Authors:** Yan Lu, Wanpeng Xi, Xiaobo Ding, Shengjie Fan, Yu Zhang, Dong Jiang, Yiming Li, Cheng Huang, Zhiqin Zhou

**Affiliations:** 1 College of Horticulture and Landscape Architecture, Southwest University, Chongqing 400716, China; E-Mails: lyttkx@gmail.com (Y.L.); xwp1999@zju.edu.cn (W.X.); dingxiaobo9@163.com (X.D.); 2 School of Pharmacy, Shanghai University of Traditional Chinese Medicine, Shanghai 201203, China; E-Mails: holyshengjie@gmail.com (S.F.); zhangyu88315@hotmail.com (Y.Z.); ymlius@163.com (Y.L.); 3 Key Laboratory of Horticulture Science for Southern Mountainous Regions, Ministry of Education, Chongqing 400715, China; 4 Citrus Research Institute, Chinese Academy Agricultural Science, Chongqing 400712, China; E-Mail: jiangdong@cric.cn

**Keywords:** citrange fruit extracts, obesity mice, metabolic disorder, regulation

## Abstract

Obesity is becoming one of the global epidemics of the 21st century. In this study, the effects of citrange (*Citrus sinensis × Poncirus trifoliata*) fruit extracts in high-fat (HF) diet-induced obesity mice were studied. Female C57BL/6 mice were fed respectively a chow diet (control), an HF diet, HF diet supplemented with 1% *w*/*w* citrange peel extract (CPE) or 1% *w*/*w* citrange flesh and seed extract (CFSE) for 8 weeks. Our results showed that both CPE and CFSE regulated the glucose metabolic disorders of obese mice. In CPE and CFSE-treated groups, the body weight gain, blood glucose, serum total cholesterol (TC) and low density lipoprotein cholesterol (LDL-c) levels were significantly (*p* < 0.05) reduced relative to those in the HF group. To explore the mechanisms of action of CPE and CFSE on the metabolism of glucose and lipid, related genes’ expressions in liver were assayed. In liver tissue, the expression level of peroxisome proliferator-activated receptor γ (*PPARγ*) and its target genes were down-regulated by CPE and CFSE supplementation as revealed by qPCR tests. In addition, both CPE and CFSE decreased the expression level of liver X receptor (LXR) α and β, which are involved in lipid and glucose metabolism. Taken together, these results suggest that CPE and CFSE administration could ameliorate obesity and related metabolic disorders in HF diet-induced obesity mice probably through the inhibition of *PPARγ* and LXRs gene expressions.

## 1. Introduction

It was estimated that there are about 1.4 billion adults who are overweight and potentially suffering from obesity [1]. Obesity is a serious health condition caused by metabolic disorders. It is a major risk factor for many chronic diseases such as type 2 diabetes, cardiovascular diseases (CVD), dyslipidemia, hypertension and some cancers [2–4]. Plant bioactive compounds were proved to be effective in the prevention and treatment of obesity and related metabolic disorders [5–7]. In the past years, active plant components and their health promotion functions have become the focus of multidisciplinary studies, and the results obtained also suggested that dietary intervention could be an effective management strategy for overweight and obesity-related metabolic disorders [8,9].

Citrus is one of the most important fruit crops worldwide and is rich in nutrients and bioactive compounds. Citrus fruits contain not only basic nutrient compounds such as vitamins, minerals, pectins and dietary fibers, but also ample bioactive compounds including flavonoids, carotenoids, limonoids and coumarins. Recently, the study of bioactive compounds has become the focus in both epidemiology and food science [10,11]. Citrus fruits have been reported to exhibit important bioactivities, including antioxidant, anti-inflammatory, anti-obesity, anti-cardiovascular and antitumor abilities [12–18]. Moreover, citrus peels have also been commonly used as culinary seasonings, food supplements and preserves in China for centuries. In recent years, the study on the use of citrus fruits in the prevention and treatment of obesity and its related metabolic diseases has attracted increasing attention. Jung *et al.* reported that *Citrus unshiu* peel extract regulated the lipid and triglyceride accumulation in 3T3-L1 adipocytes [19]. The *in vitro* lipolysis property of the *Citrus unshiu* extract was also reported [20]. In an animal experiment, Bok *et al*. found that tangerine peel extracts reduced the plasma and hepatic cholesterol levels of rats [21]. The immature *Citrus sunki* peel extract was reported to have anti-obesity effect by increasing β-oxidation and lipolysis in the adipose tissue of HF diet-induced obesity mice [22]. In addition, in our previous study, *Citrus ichangensis* peel extract was found to have anti-metabolic disorder effects in high-fat diet-induced C57BL/6 mouse [23].

More evidence suggested that citrus flavonoids play their roles through the peroxisome proliferator-activated receptors (PPARs) pathway [24]. It is well known that PPAR is the nuclear receptor transcription factor that regulates the carbohydrate and lipid metabolism of tissues and cells [25]. There are three isoforms in the PPAR family, PPARα, PPARγ and PPARδ/β, among which PPARγ mainly regulates adipocyte differentiation, lipogenesis and glucose metabolism [26,27]. The inhibitory effects of citrus flavonoids on adipogenesis and adiposity have been partially attributed to regulation of the PPAR expression levels both in cell and animal models [28,29]. It has been proved that citrus polymethoxylated flavones ameliorate lipid and glucose homeostasis and increase insulin sensitivity by regulating *PPARα* and *PPARγ* expression [30–32]. Sharma *et al*. reported that citrus naringin reduced serum lipid through up-regulating the expression of *PPARγ* and down-regulating the expression of LXRs (liver X receptor) in the liver tissue of type 2 diabetic rats [33]. In addition, the grapefruit flavonoid naringenin has been suggested as a partial antagonist of LXRα [24].

Although citrus fruits have long been utilized as foods and medicine, studies on the function and utilization of non-cultivated species remain insufficient. The plant of the genus *Citrus* L. is a rich source for foods and medicine; however, many citrus species are still under-utilized. Citrange is a generic hybrid between *Poncirus trifoliate* Raf. and *Citrus senesis* Osbeck. It is known for its well-developed root system being highly resistant to cold and pathogens. In horticulture, citrange was mainly used as rootstock for citrus variety. In previous studies, both parents of citrange, *P. trifoliata* and *C. senesis*, have been shown to have abundant bioactive compounds, especially flavonoids [34,35]. In this study, whether long-term supplementation of citrange fruit extracts (CPE and CFSE) would have a role in the prevention and treatment of obesity and its related metabolic diseases was investigated. Mice fed with HF diet and supplemented with CPE and CFSE were measured for body weight gain, glucose and lipid metabolism and the related gene expression.

## 2. Results

### 2.1. Flavonoid Contents in CPE and CFSE

**Figure 1.**
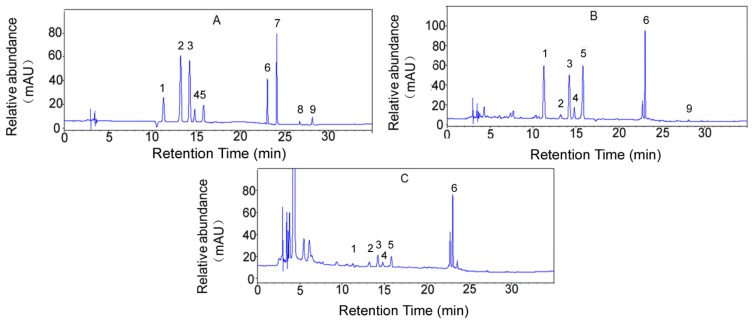
HPLC chromatograms of major flavonoids of citrange fruits extracts. The components and their relative abundance of the extracts were determined by comparison with the standard chromatogram. (**A**) The standard chromatogram: (1) neoeriocitrin; (2) narirutin; (3) naringin; (4) hesperidin; (5) neohesperidin; (6) poncirin; (7) naringenin; (8) nobiletin; (9) tangeretin; (**B**) The major flavonoid components of CPE: (1) neoeriocitrin; (2) narirutin; (3) naringin; (4) hesperidin; (5) neohesperidin; (6) poncirin; (9) tangeretin; and (**C**) The major flavonoid components of CFSE: (1) neoeriocitrin; (2) narirutin; (3) naringin; (4) hesperidin; (5) neohesperidin; (6) poncirin.

The flavonoid compositions of citrange fruit ethanol extracts (both CPE and CFSE) were analyzed using HPLC. As indicated in Figure 1, the flavonoids detected in citrange fruits mainly included neoeriocitrin, narirutin, naringin, hesperidin, neohesperidin, poncirin, naringenin, nobiletin and tangeretin. Neohesperidin (21.1 mg/g), neoeriocitrin (14.5 mg/g), poncirin (14.1 mg/g) and naringin (8.12 mg/g) were the dominant flavonoids in CPE, while CFSE mainly contained poncirin (4.85 mg/g), neohesperid in (1.87 mg/g) and naringin (0.87 mg/g). Neohesperidin and poncirin were reported to be main flavonoids in *P. trifoliate* (one of the parents of citrange) and these bioactive compounds exhibit anti-inflammatory ability [35], inhibit adipocyte differentiation and attenuate gastric disease [36,37]. Naringin, the glycoside form of naringenin, which has been reported to provide a wide range of health values including antioxidant, anti-inflammatory and anti-cancer effects in previous studies, is also abundantly presented in citrange fruits [38–42].

### 2.2. Body Weight Gain

To investigate the effects of CPE and CFSE on the metabolic dysfunction, the female C57BL/6 mice were fed respectively with a chow diet (Chow), an HF diet, HF + CPE, and HF + CFSE for eight weeks.

**Figure 2.**
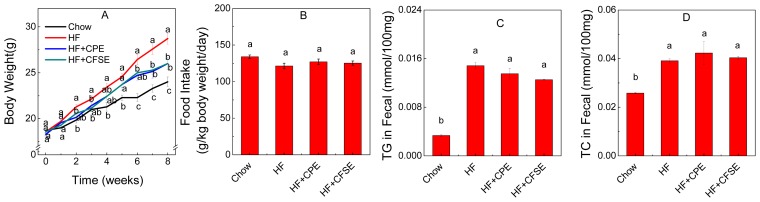
CPE and CFSE prevent HF diet-induced weight gain. C57BL/6 mice were fed respectively with a chow diet (Chow), an HF diet, HF diet supplemented with 1% *w*/*w* CPE (HF + CPE) or HF diet supplemented with 1% *w*/*w* CFSE (HF + CFSE) for eight weeks. (**A**) Body weight change; (**B**) Food intake expressed as g/kg body weight/day; (**C**) TG contents in fecal; (**D**) TC contents in fecal. The values shown are means ± SE (*n* = 7), ^a,b,c,ab^mean values with different letters were significantly different (*p* < 0.05). HF: high-fat diet; HF + CPE: high-fat diet supplemented with citrange peel extract; HF + CFSE: high-fat diet supplemented with citrange fruit and seed extract.

The final average mice body weight of HF group was significantly higher than that of Chow group (*p* = 0.00002), indicating that the high-fat diet had induced obesity (Figure 2A). CPE and CFSE supplement diet significantly decreased the body weight gain of HF diet-induced mice by 13% at the end of treatment (*p* = 0.012, 0.012). Average food intake of mice (g/kg body weight/day) in HF + CPE (*p* = 0.35) and HF + CFSE (*p* = 0.41) groups was equal to that of HF group (Figure 2B). In addition, there was no difference of TC and TG level in the collected fecal samples between the HF + CPE (*p* = 0.084), HF + CFSE (*p* = 0.067) groups and HF group (Figure 2C,D). These results suggested that citrange fruit extracts did not affect the lipid accumulation or promote lipid excretion in preventing diet-induced obesity.

### 2.3. CPE and CFSE Improves Glucose Tolerance

**Figure 3.**
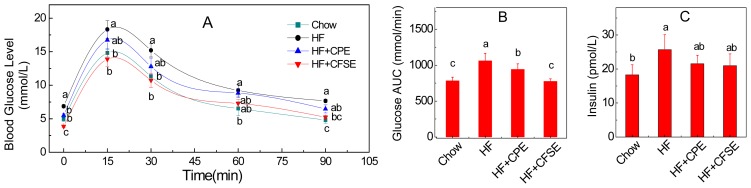
CPE and CFSE regulate the blood glucose and insulin level. (**A**) Glucose tolerance test was performed by intraperitoneal injection of glucose (1 mL/kg body weight) into mice and blood glucoses were measured at 0, 15, 30, 60 and 90 min; (**B**) Area under the curve (AUC); and (**C**) Plasma insulin level. Values are means ± SE (*n* = 7). ^a,b,c,ab,bc^mean values with different letters were significantly different (*p* < 0.05). HF: high-fat diet; HF + CPE: high-fat diet supplemented with citrange peel extract; HF + CFSE: high-fat diet supplemented with citrange fruit and seed extract.

To investigate the effect of CPE and CFSE on the metabolic disorders, the serum biochemical parameters in the mice were analyzed. Fasting blood glucose was significantly increased in the HF group mice compared to that of the chow group mice (*p* = 0.003). The CPE (*p* = 0.01) and CFSE (*p* = 0.00004) supplement groups, by contrast, showed a significant decrease in fasting blood glucose contents compared to the HF group. We also did the ipGTT examination. Figure 3A indicated that, after injection of glucose, during a 15–90 interval min, the blood glucose concentrations of HF + CPE and HF + CFSE groups remained at a level approximately 5%–25% lower than that of the HF group. The total area under the curve (AUC) of blood glucose levels between 0 to 90 min was 1059.8 ± 107.9 mmol/min for the HF group and 942.3 *±* 76.2, 775.0 ± 35.3 mmol/min for the supplement diet groups (*p* = 0.02, 0.0003), respectively (Figure 3B). These findings are similar to our previous work on *Citrus ichangensis* peel extract, in which the extract significantly reduced blood glucose level and improve glucose tolerance [23]. These results suggested that citrange fruit extracts regulated the glucose tolerance induced by HF diet in the mouse.

The supplement of citrange fruit extracts did not increase the insulin content in mouse serum compared to the HF diet (Figure 3C). Furthermore, the insulin resistance was evaluated by the homeostasis model assessment of insulin resistance (HOMA-IR) method as described previously [42]: HOMA-IR = [fasting insulin (μU/mL) × fasting glucose (mmol/L)]/22.5. The result showed that the CFSE treatment decreased the average HOMA-IR by nearly 60%, indicating CFSE has the potential to improve insulin resistance.

### 2.4. CPE and CFSE Prevents Lipid Accumulation

**Figure 4.**
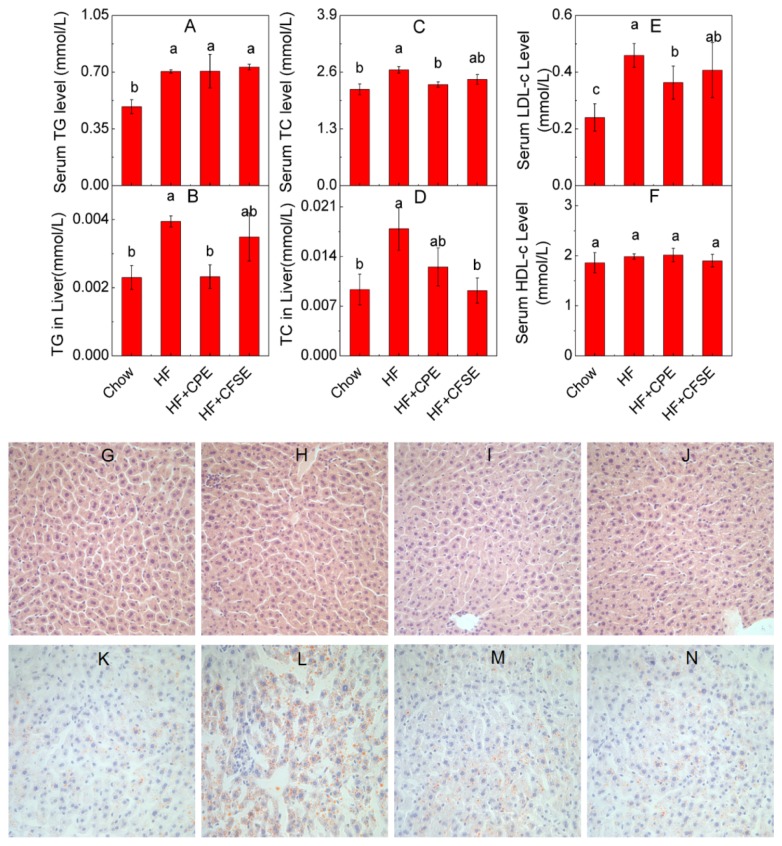
CPE and CFSE regulate lipid levels. (**A**) serum total cholesterol; (**B**) liver total cholesterol; (**C**) serum triglyceride; (**D**) liver triglyceride; (**E**) serum LDL cholesterol and (**F**) serum HDL-cholesterol; (**G**–**I**) H and E staining (×400) of livers from the (**G**) chow diet, (**H**) HF, (**I**) HF + CPE and (**J**) HF + CFSE mice; (**K**–**N**) Oil red O staining (×400) of the livers from the (**K**) chow diet, (**L**) HF, (**M**) HF + CPE and (**N**) HF + CFSE mice. The values shown are means ± SE (*n* = 6). ^a,b,c,ab^mean values with different letters were significantly different (*p* < 0.05). HF: high-fat diet; HF + CPE: high-fat diet supplemented with citrange peel extract; HF + CFSE: high-fat diet supplemented with citrange fruit and seed extract.

The fasting serum TG, TC, and LDL-c concentrations of HF diet mice were respectively elevated by 44.4%, 20.3% and 91.1% compared to those of the chow group (Figure 4A,C,E), whereas the level of HDL-c had no significant difference between mice in the HF group and chow group (Figure 4F). In addition, the TC (*p* = 0.004) and LDL-c (*p* = 0.042) levels were notably reduced by CPE treatment compared to those in the HF group, while CFSE treatment showed no significant effect on TC (*p* = 0.12) and LDL-c (*p* = 0.36) contents (Figure 4B,C). However, no difference of TG and HDL-c levels was observed between the HF, HF + CPE and HF + CFSE groups (Figure 4A,F). These findings suggest that a CPE-supplemented diet is effective in improving high-fat diet-induced dyslipidemia in mouse serum.

The liver which is sensitive to insulin plays an important role in glucose and lipid metabolism [43,44]. Therefore, the liver fat content and lipid profile of the mice were examined. The H and E staining results showed that mice fed the HF diet for 8 weeks had similar morphologies of hepatocyte tissues to the chow diet-fed mice (Figure 4G–J). Oil red O staining showed lipids were accumulated in the liver of mice of the HF group when compared to that in the chow group (Figure 4K,L), and CPE and CFSE treatments notably prevented the lipid accumulation in liver tissue (Figure 4M,N). The liver TG and TC contents were also analyzed. TC level in liver was significantly reduced in the HF + CFSE group compared to that of the HF group (*p* = 0.002). Supplementation of CPE also decreased the liver TG significantly (*p* = 0.014) (Figure 4B,D). These results suggested that citrange fruit extracts could inhibit the lipid accumulation induced by HF diet in mouse liver.

### 2.5. Gene Expression Analysis by Real-Time PCR

**Figure 5.**
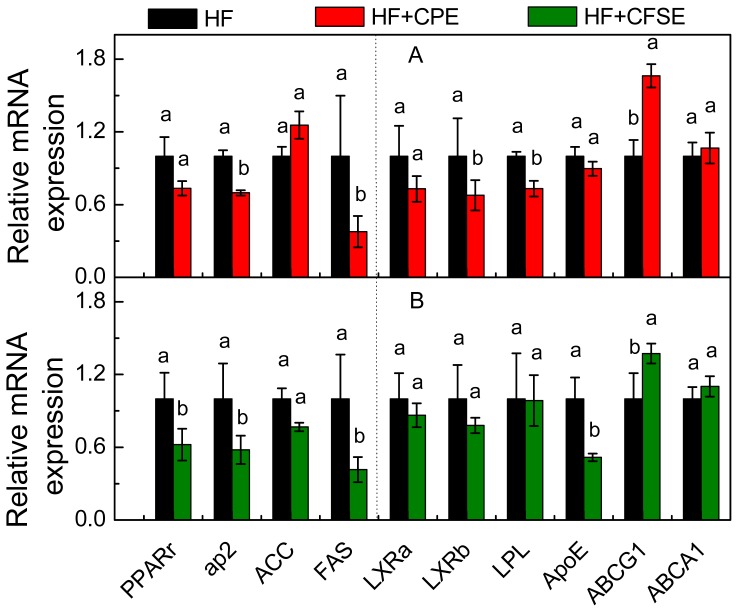
Effects of CPE and CFSE on the relative mRNA expression level of the genes in liver tissue. Beta-actin was used as an internal control. Values are expressed as means ± SE (*n* = 6). ^a,b^mean values with different letters were significantly different within the same mRNA (*p* < 0.05). HF, high-fat diet; HF + CPE, high-fat diet supplemented with citrange peel extracts; HF + CFSE, high-fat diet supplemented with citrange fruit and seed extracts.

To understand the mechanism of action of CPE and CFSE in improving hyperglycemia and dyslipidemia, the mRNA levels of *PPARγ* and its target genes including *aP2* (adipocyte fatty-acid-binding protein), ACC (acetyl-CoA carboxylase) and *FAS* (fatty acid synthase) were analyzed in the liver tissue as shown in Figure 5A. In contrast to the HF group, the *PPARγ* expressions in liver tissue in both HF + CPE and HF + CFSE groups were significantly down-regulated, especially in CFSE treatment group (*p* = 0.008). In addition, the mRNA expression levels of several PPARγ target genes in liver, including *aP2* and *FAS* were also significant down-regulated in HF + CPE and HF + CFSE groups compared to those of the HF group. Meanwhile, no significant difference was found in ACC expression between HF + CPE, HF + CFSE, and HF groups. These results suggested that CPE and CFSE could improve the lipid and glucose homeostasis by partly regulating the expression of *PPARγ* and its target genes.

Also, the mRNA expression of LXRs and its target genes involved in the synthesis of fatty acids and cholesterol, and metabolism of glucose, such as *ApoE* (apolipoprotein E), *LPL* (lipoprotein lipase), *ABCG1* (ATP-binding cassette transporter G1), and *ABCA1* (ATP-binding cassette transporter A1) in the liver tissue were also analyzed in high-fat diet groups. As shown in Figure 5B, compared to those of HF group, CPE significantly decreased the expression levels of *LXRβ* (*p* = 0.01) and *LPL* (*p* = 0.0006), while CFSE decreased that of *ApoE* (*p* = 0.0002). Both CPE and CFSE treatments significantly up-regulated the expression of *ABCG1* (*p* = 0.006, 0.01) compared to that of HF treatment. These results suggested that CPE and CFSE-supplemented diet may attenuate metabolic dysfunction partly through LXR signaling.

## 3. Discussion

Plant flavonoids are a class of secondary metabolites which may confer important health benefits such as improved regulation of glucose and lipid metabolism [45]. Citrus fruits contain an abundance of flavonoids including naringin, hesperidin, rutin, quercetin and various polymethoxylated flavones [45]. In this study, we found that the main flavonoids in CPE were neohesperidin, neoeriocitrin, poncirin and naringin, while in CFSE, there were poncirin, neohesperidin and naringin. This flavonoid composition is different from that of *Citrus ichangensis* peel extract, which mainly contained naringin, hesperidin and poncirin [23]. Previous studies indicated that naringin, poncirin and neohesperidin exhibited numerous bioactive and pharmaceutical activities, such as anti-inflammatory, antioxidant, anticancer activities and inhibiting hyperglycemia, dyslipidemia, and hepatic steatosis in type 2 diabetes [6,46–48]. According to Jung *et al*., supplementation of naringin in daily meal lowered the plasma lipids and enhanced erythrocyte antioxidant enzyme activities in hypercholesterolemic volunteers [11]. Poncirin was suggested to inhibit adipocyte differentiation in mesenchymal stem cells and prevent inflammation by inhibition of PGE2 and IL-6 production [37]. In our study, mice fed with HF diet alone showed obvious body weight gain, increases of blood glucose level, liver TG and TC levels compared to those of the chow group. We found that the symptoms caused by HF diet were alleviated by citrange fruit extract supplement treatments. In addition, CPE significantly reduced the serum TC and LDL-c level compared to those of HF group, while CFSE failed to show the same roles. These differences might attribute to the flavonoid composition and content differences in CPE and CFSE.

The specific mechanism by which citrange extracts prevent the weight gain of HF diet-fed mice is yet to be clarified. Weight gain is usually the result of an increase in adipocyte number and mass, which is caused by excess calorie intake and then stored as TG [49]. As a reduction in food intake may significantly affect body weight, one might hypothesize that the weight loss caused by CPE and CFSE supplement may result from the reduction of food intake. However, no significant difference in the amount of food intake was observed between the HF, HF + CPE and HF + CFSE groups. In addition, our data showed that CPE and CFSE had no inhibitory role on the lipids absorption. Based on these results, we suggested that the prevention functions of CPE and CFSE against body weight gain are independent of the reduction of energy intake and the absorption of lipids in mouse intestine.

PPARγ plays an essential role in lipid and glucose homeostasis [26,27]. PPARγ is a key nuclear receptor transcription factor in adipogenesis and lipogenesis. It regulates the expressions of the genes related to fatty acid oxidation, synthesis and adipogenesis, such as *ACC*, *FAS* and *aP2*. Antagonists of PPARγ have been proved to be effective in prevention and treatment of HF diet-induced obesity [50]. Gong *et al*. reported that inhibiting PPARγ activity can suppress adipocyte differentiation *in vitro* [51]. Our results indicated that CFSE significantly down-regulated the *PPARγ* gene expression in mouse liver. The expressions of *FAS* and *aP2* were also obviously suppressed in the liver of mice in HF + CPE and HF + CFSE groups. This evidence suggests that citrange fruit extracts may decrease lipogenesis partly by regulating PPARγ signaling.

It is well known that LXRs can regulate fatty acid, cholesterol and glucose homeostasis [52]. In previous studies, naringenin has been reported to be a partial antagonist of LXRα [24], suggesting that citrus flavonoids may inhibit the expression of LXRs. In this study, CFSE significantly inhibited *LXRβ* transactivities in mouse liver. In addition, we analyzed the expressions of LXRs target genes such as *ApoE*, *LPL*, *ABCG1*, and *ABCA1*. The results showed that CPE significantly decreased the expression levels of *LXRβ* and *LPL*; meanwhile CFSE decreased that of *ApoE*. We also found that the expression of *ABCG1* was significantly up-regulated by both CPE and CFSE treatment compared to that of HF treatment alone. These results suggest that citrange fruit extracts improve cholesterol and glucose metabolism partly through inhibition LXRs expression in high-fat diet-induced obesity mice.

## 4. Experimental Section

### 4.1. Preparation of Citrange Fruit Extracts

Citrange was collected from the Citrus Research Institute, Chinese Academy of Agricultural Sciences, Chongqing, China. There were 4 L of 95% ethanol added to one kilogram of fresh, sliced citrange peel and citrange flesh and seed, respectively. The mixture was extracted at 85 °C for 2 h, cooled, and then filtered with medical absorbent gauze. The filtrate were condensed at 40 °C with a rotary evaporator under reduced pressure, freeze-dried to a powder, and kept at −20 °C until use. The frozen dried powder of CPE and CFSE was used as HF-induced mice supplemented diet.

### 4.2. HPLC Analysis

For the frozen dried powder of CPE and CFSE, 0.1 g was dissolved in 50 mL methanol then filtered with 45 μm filter membrane preparing for the samples for HPLC. The flavonoids content of CPE and CFSE were determined at 280 nm by using Agilent 1200 liquid chromatography system (Agilent Technologies, Santa Clara, CA, USA) equipped with a FID detector and a C18 Column (250 × 4.6 mm, 5 μm, Agilent Technologies, Santa Clara, CA, USA). The column was operated at 30 °C, and 10 μL extract was injected into HPLC. The mobile phase consisted of 100% acetonitrile (A) and water containing 0.5% acetic acid (B) at a flow rate of 1.0 mL/min. The gradient profile was as follows: 0–12 min, 85%–75% B; 12–17 min, 75% B; 17–20 min, 75%–50% B; 20–30 min, 50%–25% B; 30–35 min, 25%–5% B; and 35–40 min, back to 85% B. The standards (purity > 98%) were purchased from Shanghai R & D Center for Standardization of Chinese Medicines (Shanghai, China).

### 4.3. Animals and Diets

Six-week-old C57BL/6 female mice were purchased from the SLAC Laboratory (Shanghai, China). The animal study protocols were approved by the Shanghai University of Traditional Chinese Medicine (Approval number: 12006). The mice were kept under 22–23 °C on a 12 h light/dark cycle for one week to adapt. After adaptation period, C57BL/6 mice were weighed and randomly separated into four groups (seven mice/group): chow diet (10% of calories derived from fat, Research Diets; D12450B; New Brunswick, NJ, USA), HF diet (60% of calories derived from fat, New Brunswick, NJ, USA, Research Diets; D12492), HF diet supplemented with 1% *w*/*w* CPE (HF + CPE) and HF diet supplemented with *w*/*w* CFSE (HF + CFSE) for 8 weeks (each group of mice were housed in one cage). The average flavonoid dose given to mice in the HF + CPE group was 72 mg/kg body weight/day, or 9.3 mg/kg body weight/day in group HF + CFSE. Food intake and body weight were measured every other day. The mice were given free access to food and water.

### 4.4. Intraperitoneal Glucose Tolerance Test (ipGTT)

After a 12 h fast, basal blood glucose levels (0 min) were determined from the tail vein of all mice using Accu-Chek blood glucose monitoring (Roche, Mannheim, Germany). The mice were then given an intraperitoneal injection with 20% glucose (1 mL/kg body weight) and the blood glucose subsequently measured at 15, 30, 60 and 90 min. The glucose tolerance test area under the curve (AUC) was calculated according to the previous study [53], by using the formula termed “area under the curve respect to ground”.

### 4.5. Serum Chemistry Analysis

After 12 h of fasting, all mice were anesthetized with urethane before collecting blood samples. Blood samples were collected from the heart by using EDTA-coated tubes and were then allowed to clot at room temperature for 30 min.

Serum samples were separated from the blood after centrifugation. Serum lipid parameters of triglyceride (TG), total cholesterol (TC), low density lipoprotein cholesterol (LDL-c), and high density lipoprotein cholesterol (HDL-c) were determined using a Hitachi 7020 Automatic Analyzer (Hitachi, Tokyo, Japan). Serum lipid parameters were measured enzymatically by Clinical Reagents according to the manufacturer’s procedure.

### 4.6. Liver and Fecal Lipid Content Analysis

After collecting the blood sample, epididymal fat and liver were collected, weighed, snap-frozen using liquid nitrogen and stored at −80 °C for further experiments. Of the frozen liver tissue, 50 g was minced and homogenized in 1 mL of tissue lysis buffer (20 mM Tris·HCl, pH 7.5, 150 mM NaCl, 1% Triton) and mixed with an equal volume of chloroform. The chloroform layer was separated, dried, and re-suspended in 100 μL of isopropyl alcohol to measure the lipid levels as described above. Fecal lipids were extracted and measured as described above.

### 4.7. Histology of Liver

Frozen liver samples were embedded in OCT (Sakura Finetek, Torrance, CA, USA) and sliced into 8 μm sections for hematoxylin and eosin (H and E) and oil red O (Sigma, St. Louis, MO, USA) staining. Scanning electron microscopy was used to examine the structure of fat tissue according to the previously described protocols [54]. The images were taken using a Philips XL-30 scanning electron microscope (Philips, Eindhoven, The Netherlands).

### 4.8. RNA Isolation and Gene Expression

Total RNA in liver was extracted by a spin column (Qiagen, Hilden, Germany) following the manufacturer’s protocol. The first-strand cDNA was synthesized using the cDNA synthesis kit (Fermentas, Madison, WI, USA). The gene expression levels were determined by quantitative real-time RT-PCR conducted using the ABI Step One Plus Real-Time PCR system (Applied Biosystems, Carlsbad, CA, USA). The primers involved in the experiments were shown in Table 1. The cDNA was denatured at 95 °C for 10 min followed by 40 cycles of PCR (95 °C, 15 s, 60 °C, 60 s). All results were obtained from at least three independent experiments. Beta-actin was used as an internal control to normalize differences in template amounts.

### 4.9. Statistical Analysis

All data were obtained from at least three independent experiments and expressed as the mean ± SE unless otherwise indicated. All data were analyzed by the SPSS package (Version 13.0, SPSS, Chicago, IL, USA). One-way ANOVA model and Duncan’s multiple range tests at 5% level were used to determine the significance of differences between groups. Origin Pro 8.0 SR4 (Origin Lab, Northampton, MA, USA) were used to make graphs.

## 5. Conclusions

In the present study, neohesperidin, neoeriocitrin, poncirin and naringin were found to be the main flavonoid components in CPE and CFSE. Supplementation of CPE prevented the weight gain and reduced serum glucose, TC and liver TG level of HF-induced obesity mice, inhibited the mRNA expression of *ap2*, *LXRβ* and *LPL* in mouse liver; while supplementation of CFSE reduced mouse weight, serum glucose, serum LDL-c and liver TC level, showing inhibition of the mRNA expression of *PPARγ*, *ap2*, *FAS* and *ApoE* in mouse liver. These results indicated that CPE and CFSE may alleviate the obesity-related metabolic dysfunction in HF-induced mice partly through the PPARγ and LXRs pathways. Among the main flavonoids of CPE and CFSE, naringin and poncirin may be the possible key bioactive compounds in this process. However, further studies are essential to the identification of the key bioactive compound(s) of citrange fruit and systematically reveal their mechanism of action involved in glucose-metabolism and adiposity.

## Figures and Tables

**Table 1.** Sequences of the primers used in real-time PCR.

Gene	Forward primer	Reverse primer
*β-Actin*	TGTCCACCTTCCAGCAGATGT	AGCTCAGTAACAGTCCGCCTAGA
*PPARγ*	CGCTGATGCACTGCCTATGA	AGAGGTCCACAGAGCTGATTCC
*aP2*	CATGGCCAAGCCCAACAT	CGCCCAGTTTGAAGGAAATC
*ACC*	GAATCTCCTGGTGACAATGCTTATT	GGTCTTGCTGAGTTGGGTTAGCT
*FAS*	CTGAGATCCCAGCACTTCTTGA	GCCTCCGAAGCCAAATGAG
*LXRα*	GAGTGTCGACTTCGCAAATGC	AGCTCAGTAACAGTCCGCCTAGA
*LXRβ*	CAGGCTTGCAGGTGGAATTC	ATGGCGATAAGCAAGGCATACT
*ApoE*	GAACCGCTTCTGGGATTACCT	TCAGTGCCGTCAGTTCTTGTG
*LPL*	ATCGGAGAACTGCTCATGATGA	CGGATCCTCTCGATGACGAA
*ABCG1*	TCCCCACCTGTAAGTAATTGCA	TCGGACCCTTATCATTCTCTACAGA
*ABCA1*	GGCAATGAGTGTGCCAGAGTTA	TAGTCACATGTGGCACCGTTTT

Note: The gene names in the paper are shown. *β-Actin*, beta-actin; *PPARγ*, peroxisome proliferator-activated receptor gamma; *aP2*, adipocyte fatty-acid-binding protein; *ACC*, acetyl-CoA carboxylase; *FAS*, factor associated suicide; *LXRα*, liver X receptor α; *LXRβ*, liver X receptor β; *ApoE*, apolipoprotein E; *LPL*, lipoprotein lipase; *ABCG1*, ATP-binding cassette transporter G1; *ABCA1*, ATP-binding cassette transporter A1.
